# Single-center experience of surgically resected gastrointestinal stromal tumors: A report of six cases, including a rare case involving the lower esophagus

**DOI:** 10.3892/ol.2014.2792

**Published:** 2014-12-11

**Authors:** BILAL O. AL-JIFFRY, HISHAM M. ALLAM, MOHAMMED HATEM

**Affiliations:** 1 Department of Surgery, Taif University, Taif 21944, Saudi Arabia; 2 Department of Surgery, Al-Hada Military Hospital, Taif 21944, Saudi Arabia

**Keywords:** gastrointestinal stromal tumors, esophagus, imatinib

## Abstract

Gastrointestinal stromal tumors (GISTs) are rare, but remain the most common GI mesenchymal neoplasms. In the present study, six cases of GIST are reported, and one of these cases, a patient with esophageal GIST, is reported in-depth. Certain recent developments in the clinical therapy of GISTs are also discussed. The records of all surgically-resected GI stromal tumors treated at the Al-Hada Military Hospital between January 2007 and December 2012 were reviewed. There were six cases of surgically resected GISTs during this time period, three males and three females, with a mean age of 69.3±16.4 years. The stomach was involved in 66.7% of cases, the small intestine in 16.7% and the esophagus, which is an extremely rare site, in 16.7% of cases. The most common symptom at presentation was abdominal pain, followed by GI bleeding. The mean tumor size was 8.7±6.3 cm. Surgery was indicated by the presence of the aforementioned symptoms or a tumor size >5 cm. All tumors were completely resected with histologically negative margins. The diagnoses were established by immunohistochemistry. Four patients were classified as possessing a high-grade variant, and were administered with tyrosine kinase inhibitors (TKIs). Following a mean follow up of 31 months, no recurrence or mortality was detected. Complete surgical resection with tumor-free margins is the standard treatment for GISTs, and TKIs should be used as adjuvant therapy if the risk of progressive disease is high.

## Introduction

Gastrointestinal (GI) mesenchymal neoplasms are rare tumors categorized into two groups, with GI stromal tumors (GISTs) being the most common. However, GISTs only account for <1% of all GI tumors (1,2). The exact incidence rate of GISTs is unknown, as certain resected tumors are not examined genetically and minute tumors, termed GIST tumorlets, are generally not included in cancer registries. However, the annual age-adjusted incidence in the US has been estimated to be 6.8 cases per million individuals (3). In other studies, the reported prevalence of GIST varies between 10 and 20 cases per million individuals (1–3). The mean age at presentation is 60 years, with no gender, ethnic or geographic predominance (4).

GISTs mainly occur sporadically, but rare hereditary variants, which may present as multiple primary tumors, have been reported (5). The stomach is the most common site of GISTs, accounting for 60% of cases, followed by the small intestine, particularly the proximal jejunum, and the large intestine, particularly the rectum, accounting for 30 and 5% of cases, respectively. The esophagus is affected in <1% of cases, and tumors are rarely detected in the omentum, mesentery and peritoneum (4). GISTs are positive for cluster of differentiation (CD)34 expression in ~80% of cases and positive for CD117 expression in ~95% of cases. However, certain studies have found that a diagnosis in CD117-negative cases can be made based on clinical and morphological features (6,7). The intestinal Cajal cells are considered to be the cells of origin of GISTs, as they are the only cells known to demonstrate dual positivity for CD117 and CD34 (6,7).

The number of mitotic cells per high-power field (HPF) determines the malignant potential of the tumor, and tumors are considered benign if the number of mitotic cells is less than one per HPF, potentially malignant if there are between one and five mitoses per HPF and malignant if there are more than five per HPF (8). Complete surgical resection followed by imatinib treatment remains the treatment of choice for these tumors (9).

In the present study, a case series of patients diagnosed with GISTs is presented, in addition to a case report of an esophageal variant and a discussion of the most recent developments in the clinical therapy of GIST.

## Case report

### Report of six cases

**Figure 1:**
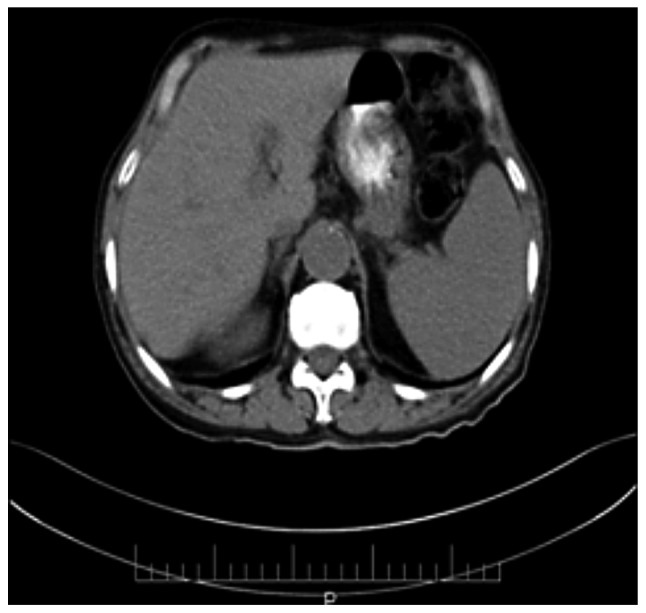
Computed tomography scan revealing a gastrointestinal stromal tumor of the stomach measuring 6×4×5 cm.

**Figure 2:**
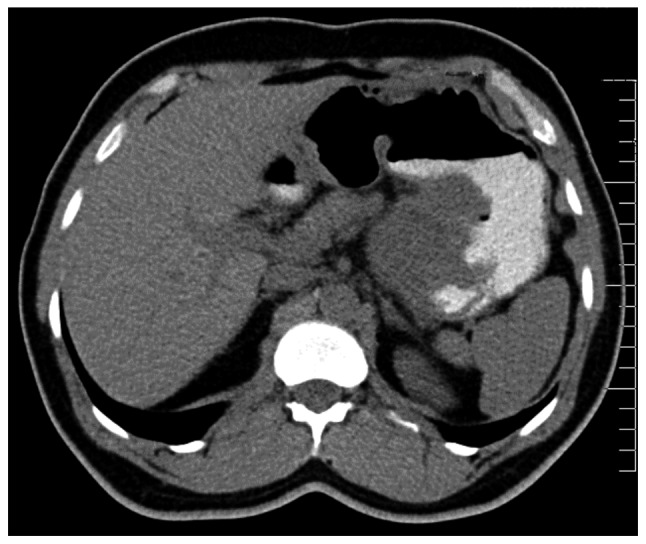
Computed tomography scan revealing an intestinal gastrointestinal stromal tumor measuring 10×15×9 cm.

**Figure 3:**
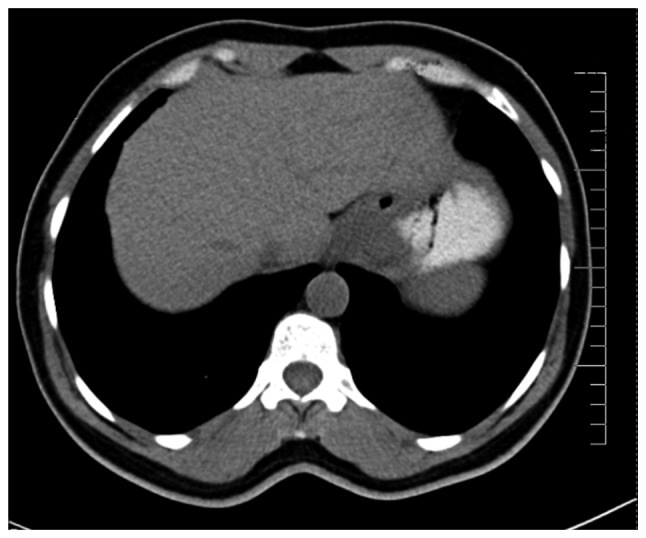
Computed tomography scan revealing an esophageal gastrointestinal stromal tumor measuring 7×5×3 cm.

**Figure 4:**
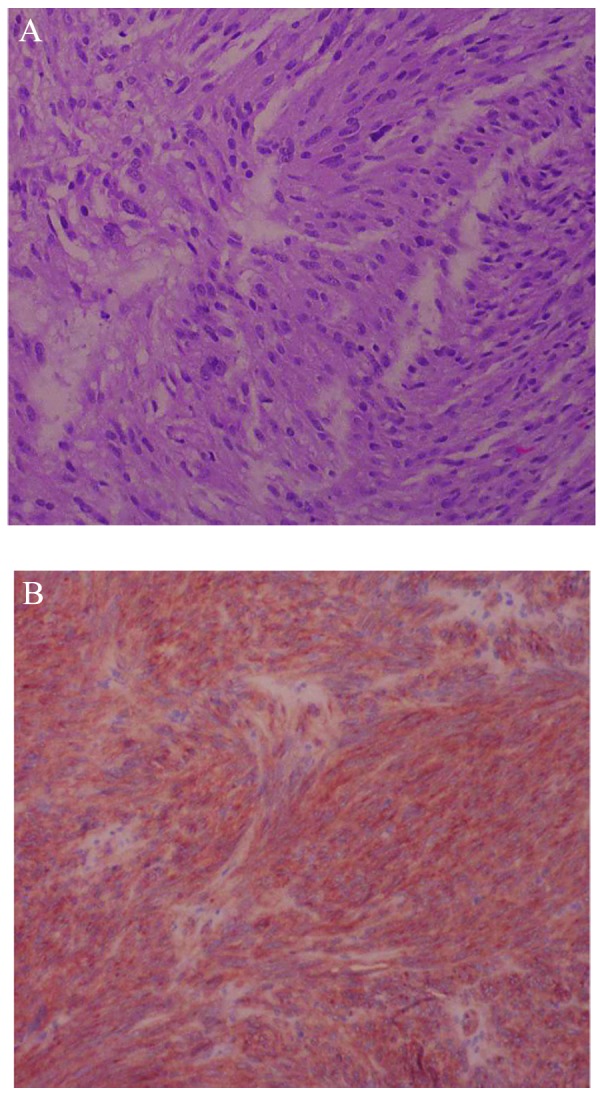
Immunohistochemical staining. (A) Hematoxylin and eosin stain of the stomach revealing a spindle cell gastrointestinal stromal tumor. (B) The same tissue specimen revealing a positive result for CD117 expression.

Between January 2007 and December 2012, six patients diagnosed with GISTs underwent surgical resection at the Al-Hada Military Hospital (Taif, Saudi Arabia). The patients consisted of three males (50%) and three females (50%). Informed consent was obtained from all patients. The mean age at presentation was 69.3±16.4 years (range, 51–80 years). In the current series, the stomach was the most common site of lesions, with four out of six patients (66.7%) possessing a GIST of the stomach, followed by one patient possessing a tumor of the small intestine (16.7%) and one patient with an esophageal tumor (16.7%), which is the focus of the present case report. The five patients with stomach and small intestine tumors underwent abdominal CT prior to surgery, and the four patients with stomach GIST also underwent upper endoscopy and biopsy. All four biopsies showed spindle cells, which were highly indicative of GIST. In addition, blood tests for all six patients were within normal limits, with the exception of two patients that exhibited microcytic hypochromic anemia. Surgery was performed for symptomatic tumors, with pain being the most common presenting symptom, occurring in four out of six patients (66.7%), followed by GI bleeding in the other two patients (33.3%). The other indication for surgery was a tumor size of ≥5 cm. The tumor sizes ranged between 4–15 cm, with a mean size of 8.7±6.3 cm. The computed tomography (CT) findings of three patients are shown in Figs. 1–3. All patients underwent curative surgical resection, and all specimens possessed histologically negative margins. Histopathological examination revealed the presence of a spindle-cell pattern in four out of six of the tumors (66.7%) and the remaining two specimens were classified as epithelioid (16.7%) and mixed type (16.7%), respectively. Five specimens (83.4%) were CD117-positive (Fig. 4), and one sample (16.7%) was CD117-negative. In five specimens, the status of CD34 was examined, and these samples were all found to be positive for the expression of CD34, while the remaining sample was not examined.

In four patients, the resected tumors were classified as GISTs with a high risk of progressive disease (66.7%; 5–34/50, 13/20, 26/50 and 31/50 mitoses per HPF, respectively), one patient was classified as having a moderate risk (16.7%; 2/20 mitoses per HPF) and the remaining patient was classified with a low risk (16.7%; 5/50 mitoses per HPF). All four patients with high-risk tumors received daily treatment with 400 mg imatinib, a tyrosine kinase inhibitor (TKI), for three years following surgery. However, the remaining two low-risk patients did not receive any further treatment. Subsequent to a mean follow up of 31±7.3 months, all patients remained disease free.

### In-depth case report of an esophageal GIST

A 51-year-old male presented to the gastroenterology clinic at Al-Hada Military Hospital at the end of February 2011 with dysphagia that had progressively developed over the previous two months and melena that had developed over the previous two weeks. The patient experienced no other GI symptoms, and the findings of a systemic review were unremarkable. The clinical examinations were normal, with the exception of a mild tenderness in the epigastric area. The results of the laboratory examinations were normal, apart from the hemoglobin and hematocrit levels, which were 10.9 mg/dl (normal range, 14–18 mg/dl) and 32.8% (normal range, 42.0–52.0%), respectively. Upper GI endoscopy revealed a gastro-esophageal mass and a biopsy of the mass revealed spindle cells, which are suggestive of GIST. A CT scan revealed a mass at the gastro-esophageal junction measuring 7 cm longitudinally, 5 cm transversely and 3 cm in the sagittal plane. Metastatic examination revealed that there was no metastasis.

The patient underwent surgery on March 5, 2011. Intra-operatively, a large tumor was found in the lower esophagus and gastro-esophageal junction. The entire tumor was removed through distal esophagectomy and proximal gastrectomy. The specimen was subsequently pathologically examined. The reported size was 7×5×3 cm, and the proximal and distal donuts were free of neoplasia. The tumor tissue revealed spindle cells, which were strongly positive for CD117 and CD34, with a mitotic rate of 13 cells per 20 high-power fields. The final diagnosis was a high-risk GIST (T3N0M0). The patient received treatment with the TKI imatinib (400 mg orally, once a day for three years) and remains recurrence-free at present.

## Discussion

The reported global prevalence of GISTs varies between 10 and 20 cases per million individuals, but the actual burden of GISTs in Saudi Arabia is not known (1–3). However, the present study reported six patients with these neoplasms that presented to Al-Hada Military Hospital, which is one of three tertiary hospitals serving approximately two million people in the Taif region of Saudi Arabia. The mean age at presentation in the current series was 69 years, which is slightly higher than that recorded in the literature (1). The most common presenting symptom was abdominal pain, followed by GI bleeding, and no metastases were recorded. Other studies have reported that GI bleeding is the most common symptom of GISTs (7).

GISTs are positive for CD117 in ~95% of cases, and it has been revealed that 70–80% of tumors are generally positive for CD34 expression (6,7). In five out of six patients in the present series, the lesions were positive for the expression of these markers, as determined by immunohistochemical analysis.

Surgery is the standard treatment of GIST, as the tumors are chemo- and radio-resistant (8,9). However, following apparently adequate surgical resection, local or distant metastases are usually recorded in approximately half of the patients (8–10). However, the prognosis has dramatically improved in recent years with the development of targeted therapy, including the TKIs imatinib and, in imatinib-resistant cases, sunitinib (1). TKIs block the ability of c-Kit and the platelet derived growth factor receptor-α proteins, thereby resulting in attenuated or reduced tumor cell growth and division (10). The use of these inhibitors as adjuvant therapy following adequate surgical resection in high-risk tumors has been demonstrated to reduce recurrence markedly (11). In the present study, adjuvant TKI therapy was used for patients who were classified as possessing GISTs with a high risk of progressive disease, whereas surgery was the only treatment for patients with a moderate or low risk. No recurrence was detected in either group subsequent to a mean follow-up period of 31±7.3 months. The long-term benefits of this type of therapy, in addition to the optimal disease risk stratification and the optimal duration of adjuvant treatment, remain unclear (12).

The esophagus is a rare site for GIST occurrence, representing <1% of all reported GISTs in the literature, and these tumors are more challenging to manage compared with those that arise in intra-abdominal organs, largely due to a lack of serosa confining the tumor (13,14). In addition, mobilization of the distal esophagus is difficult due to the segmental nature of the blood supply to the organ and thus, it may lead to blood deprivation in the left gastric and left phrenic arteries, resulting in devascularization and ischemia of the mobilized area (13). Although local resection of small tumors arising from the lower part of the esophagus is reasonable if negative resection margins can be achieved, an open en bloc esophagectomy is recommended for tumors ≥2 cm, and for those involving the gastroesophageal (GE) junction (14,15). In the present patient with an esophageal GIST, the tumor measured 7×5×3 cm and was located at the GE junction, so esophagogastrectomy was performed. The optimal treatment of esophageal tumors <2 cm in size is controversial, as certain studies recommend a follow-up using endoscopic ultrasound and subsequent excision of tumors that increase in size. However, the Canadian guidelines suggest that all GISTs must be resected, regardless of their size, to reduce the risk of metastases (8). In conclusion, complete surgical resection with negative margins is the standard treatment for GISTs, but TKIs can be used as adjuvant therapy if the risk of progressive disease is high.
